# Contextually tailored interventions can increase evidence-informed policy-making on health-enhancing physical activity: the experiences of two Danish municipalities

**DOI:** 10.1186/s12961-018-0290-4

**Published:** 2018-02-21

**Authors:** Maja Bertram, Natasa Loncarevic, Christina Radl-Karimi, Malene Thøgersen, Thomas Skovgaard, Arja R. Aro

**Affiliations:** 1 Department of Public Health, University of Southern Denmark, Unit for Health Promotion Research, Niels Bohrs Vej 9, 6700 Esbjerg, Denmark; 2 Region of Southern Denmark, Center for Quality, P V Tuxens Vej 5, 5500 Middelfart, Denmark; 3 Danish Institute for Non-Formal Education/Danish Institute for Sport Studies, Frederiksgade 78B, 8000 Aarhus C, Denmark; 4 Department of Sports Science and Clinical Biomechanics, University of Southern Denmark, Campusvej 55, 5230 Odense M, Denmark

**Keywords:** Evidence integration, Implementation research, Participatory approach

## Abstract

**Background:**

The present study aims to test out contextually tailored interventions to increase evidence-informed health-enhancing physical activity policy-making in two Danish municipalities.

**Methods:**

The study was performed as experiments in natural settings. Based on results from a pre-intervention study defining the needs and contexts of the two settings, the interventions were developed based on logical models. The interventions aimed at increasing the use of knowledge in policy-making, primarily via strengthening intersectoral collaboration. The interventions were evaluated via pre-, post- and 12-month follow-up questionnaires and qualitative interviews were carried out prior to the intervention start.

**Results:**

The use of knowledge changed in several ways. In one municipality, the use of stakeholder and target group knowledge increased whereas, in the other municipality, the use of research knowledge increased. In both municipalities, the ability to translate knowledge to local context, the political request and the organisational procedures for use of knowledge increased during the interventions. There was some variation between the two settings, which shows the importance of tailoring to context. Most of the changes were diminished at the 12-month follow-up.

**Conclusion:**

Contextually tailored interventions have the potential to increase evidence-informed policy-making on health-enhancing physical activity. However, this finding needs to be tested in larger samples and its sustainability must be strengthened.

**Electronic supplementary material:**

The online version of this article (10.1186/s12961-018-0290-4) contains supplementary material, which is available to authorized users.

## Background

Physical activity has a strong positive influence on health status [1]. At the same time, research clearly shows that structural and contextual factors, such as policies, have the greatest influence on levels of physical activity among populations [2]. However, to have maximal impact, such policies need to be informed by research evidence [3, 4]. Based on a scoping review [5], there are still clear gaps in research pertaining to physical activity policy-making. However, it has been suggested that the relevant research evidence should be divided into three categories, namely (1) studies that link physical activity to risk factors of health outcomes, (2) studies that link interventions to physical activity behaviour, and (3) studies that link policy-making to physical activity [5].

Generally, in public health and health promotion, the integration of research evidence into policy-making is more complicated than within clinical/medical areas [6]. This is mainly because research evidence is rarely the primary driver in public health. Instead, the social and political contexts, including population characteristics, values and preferences as well as political priorities and resources, need to be taken into account [3]. Hence, policy-making for health-enhancing physical activity (HEPA) should be evidence-informed – meaning that research evidence informs the policy-making process instead of dictating it.

Because HEPA policy-making is highly dependent on context, efforts to strengthen evidence-informed policy-making (EIPM) must be tailored to the specific policy being developed [7–11]. Therefore, the activities conducted in order to increase the integration of evidence into a given policy process should start with a thorough analysis of the specific needs, wishes, values and interests of key stakeholders in the development of the policy – together with other contextual factors that can be presumed to impact policy development [7, 12, 13]. Based on these considerations, contextually tailored interventions to enhance EIPM can be developed and implemented. Despite the accumulating literature suggesting contextually tailored interventions as an appropriate method for increasing EIPM, few examples of its practical implementation have been reported, and thus a knowledge gap exists [14].

The aim of this article is to present the implementation and outcomes of contextually tailored interventions in EIPM for HEPA in two Danish municipalities.

## Methods

Implementation of contextually tailored interventions constituted one component of the project REsearch into POlicy to enhance Physical Activity (REPOPA) [15]. The overall aim of REPOPA (2011–2016) was to find new ways to integrate research evidence with policy-making in the development of HEPA policies. REPOPA was a programmatic research project with three overall phases. Firstly, the challenges of doing EIPM in the area of HEPA were described via document analysis [16, 17], secondly, two different types of interventions were tested (policy games [18] and tailored interventions [11]) and, finally, a set of recommendations was developed via a Delphi approach. The main principle of REPOPA activities was that research evidence does not stand alone, but needs to be integrated with other sources of relevant and contextual knowledge [15].

One REPOPA objective was to design and test tailored interventions aimed at facilitating EIPM on HEPA in selected real-world policy environments (in Denmark, the Netherlands and Italy) as experiments in natural settings. Theoretically, these interventions were guided by elements of the Stewardship approach – an inclusive and participatory approach for the development of public health interventions. The specific elements used were close and equal collaboration between policy-makers and researchers and the importance of context and transparency [19]. In addition, key literature on tailored interventions was used as guidance [8, 10, 20–23]. It was hypothesised that such a combination of approaches could enhance close interaction between the main stakeholders – policy-makers and researchers – and thereby promote the optimal use of both formal research evidence and contextual knowledge in HEPA policy development. In this paper, we will use the term ‘research knowledge’ for research evidence, the term ‘stakeholder knowledge’ for the experiences, values, perceptions, etc. derived from internal stakeholders (such as policy-makers and municipal administrative staff) and external stakeholders (such as non-governmental organisations and local business), and the term ‘target group knowledge’ for information on the characteristics, values, wishes, etc. of the group(s) targeted by the policy.

The pre-intervention phase of the study, including detailed information on the methods for the selection of policy cases, the results of needs assessments and context analysis, and the development of intervention contents and tools for outcome measurement (questionnaires and qualitative interviews), has been presented in Bertram et al. [11]. A brief explanation of these will be given below, together with additional reflections and information on the settings, the policies and participants, the implementation process, and the data used for evaluation.

### Settings, policies and participants

In each of the three mentioned countries, two municipalities were selected as settings based on their willingness to participate and the fact that they were in a process of developing a HEPA policy. The two Danish municipalities, Kolding and Varde, are those reported on in this paper, since these were comparable in relation to the policy, context and culture themes.

Kolding and Varde started a policy development process in relation to HEPA in 2013. Varde had experience working together with university researchers in EIPM. Kolding did not have specific experience within this area but had cooperated with researchers in relation to other fields.

The interventions, being experiments in natural settings, were purposely attached to the development of the ongoing HEPA policy-making processes; they took place during the periods February 2013 tp April 2014 (Kolding) and September 2013 to May 2014 (Varde). The researchers did not control the process, including the selection of participants in the policy development process or the specific aims and contents of the policies to be developed. Rather, their role was to facilitate the use of knowledge and the collaboration between policy-makers and researchers in the policy processes in progress. The underlying policy development processes would have taken place with or without the interventions.

The participants in the interventions are presented in Table 1. The two groups differed in their composition, especially in relation to their power and function in the municipalities. In Kolding, the participants were heads of departments, meaning that they had strong formal power. In Varde, the participants were primarily consultants, meaning that they were an active part of the daily work in the departments and had close contact with practitioners.

**Table 1:** Intervention participants in HEPA policy development in two municipalities

Department	Positions
Kolding	
Social services and health (responsible for policy development)	Head of department, Health planner, Coordinator, Development manager
Environment	Head of department, Consultant
Transportation	Head of department, Engineer
Planning	Head of department, Architect, Urban planner
School and education	Head of department, Consultant
Elderly	Head of department, Project leader
Culture	Head of department, Consultant
Leisure	Head of department, Consultant
Varde	
Health and rehabilitation (responsible for policy development)	Head of department, Development consultant, Prevention consultant, Therapist
Children and youth	Youth consultant, School consultant, Sports consultant
Planning and technical services	Playground inspector
Culture and leisure	Development consultant
Centre for employment	Job consultant
Human resource management and personnel department	Human resource management consultant

### Implementation process

The needs assessments in the two municipalities displayed a need for more systematic and structured working methods in relation to finding and applying relevant knowledge in the HEPA policy development. Furthermore, a need for better collaboration between the sectors in the municipalities and with researchers was found [11]. Based on these results, the overall aim of the contextually tailored intervention in both municipalities was to build interventions that could strengthen a close interaction and collaboration between researchers, policy-makers, target groups and other relevant stakeholders. The specific aim was defined as increasing the use of knowledge (1) from research, (2) from stakeholders, and (3) of target groups to eventually promote EIPM. The focus was also on strengthening intersectoral collaboration in the municipalities and with researchers, as this was seen as a facilitator of knowledge use [16].

Both interventions were designed based on logical models (a model to clarify the theory of change) that theoretically linked intended activities with expected outcomes [24]. The specific purpose of these logical models was to explicate the assumptions behind the interventions, namely how were the different steps in the interventions expected to work and how did this relate to the final expected outcome of the intervention processes.

The intervention in Kolding consisted of four workshops, each of which had a timeframe of 4–5 h. Table 2 provides an overview of the workshops and their contents. Before each workshop, the researchers and the municipal representatives from the Social Services and Health department, who were responsible for the development of the policy, had a planning meeting. The aim of this meeting was to set the specific objectives and activities for the workshop. After completing each workshop, they again had a debriefing session, where further progress of the work on policy development was discussed [13].

**Table 2:** Overview of policy intervention contents in the two Danish municipalities

Kolding				
	Workshop I	Workshop II	Workshop III	Workshop IV
Date	29.4.2013	24.6.2013	9.10.2013	27.11.2013
Content	Facilitated discussion in groups on vision Facilitated discussion in groups on the first steps toward realising the vision of the policy	Homework: Each of the participant filled out a scheme on initiatives directly or indirectly related to physical activity in their own administrative section Facilitated discussion groups on vision Facilitated discussion on strategic focus points	Presentation of possible models for intersectoral collaboration based on a rapid evidence assessment of relevant scientific literature and knowledge from the qualitative interviews Facilitated discussion groups on the possible models for intersectoral collaboration in Kolding	Case-based group work with the aim of testing the potentials of the intersectoral network Process evaluation
Varde				
	Meeting I	Meeting II	Meeting III	Meeting IV
Date	28.10.2013	16.12.2013	13.3.2014	12.6.2014
Content	Matching of expectations for the policy Discussion of resources and barriers related to physical activity initiatives Homework: Mapping of existing activities related to physical activity	Discussion of policy template Presentation of existing health-related activities Discussion of intersectoral collaboration and relational coordination	Discussion of policy content – based on the homework of the participants Oral presentation from REPOPA with knowledge input on the different themes in the draft – combined with a short report	Discussion of policy content and idea catalogue Discussion of the inclusion of policy objectives in the municipal management style in Varde municipality Process evaluation

The intervention in Varde was based on four meetings. These differed from the workshops in Kolding by being shorter and primarily based on roundtable discussions. Except for the first meeting, which lasted 3 h, the meetings had a 1½-hour timeframe. Table 2 provides an overview of the four meetings and their contents. The meetings, based on discussions with the active participation of the working group members, were characterised by high attendance and participatory discussions. In between meetings, the participants from the Health and Rehabilitation department, who were responsible for the development of the policy, had several face-to-face and Skype meetings with the researchers. The purpose of these meetings was to plan the process and the contents of the intervention as well as the specific policy development, particularly in relation to the development of a basic structure of the policy [13].

### Data – questionnaires

To measure the intervention outcome, an internet-based questionnaire was used. The questionnaire was designed for the international project REPOPA [15], including country-specific questions, and translated to local languages. In this case, a Danish version of the questionnaire was used. The English version of the questionnaire is published as Additional file 1.

The questionnaire was developed based on areas noted as important in the needs assessments from the pre-intervention study [11] and in current literature on EIPM [4, 25]. The data reported in this paper are derived from questions related to the categories of use of knowledge (1) from research, (2) from stakeholders, and (3) on target groups. The use of knowledge was assessed in relation to (1) conceptual knowledge use (knowledge being used to enlighten) [26, 27], (2) knowledge translation, (3) request for evidence/knowledge, (4) organisational procedures (established routines or guidelines for the identification and use of research knowledge), and (5) instrumental knowledge use (knowledge being directly used in policy formulation) [26, 27]. Additionally, the questionnaire also contained questions in relation to perceived facilitators and barriers to the use of evidence and knowledge, perceived facilitators and barriers to intersectoral collaboration, and prioritisation of the importance of the different types of evidence and knowledge. Additional file 1 contains the full questionnaire, with marks on the questions used herein.

A five-point Likert scale (1: to a very low degree, 2: to a low degree, 3: neither to a low or high degree, 4: to a high degree, 5: to a very high degree), as well as a ‘do not know’ option, was used. Additionally, the respondents could add comments to their answers in open text boxes.

The first measurement was conducted before the intervention (year 2013), one measurement right after the intervention (year 2014) and one measurement 12 months after the intervention (year 2015) [11].

The response rates varied, with the highest rate in the pre-intervention measurement (Kolding in May–April 2013, n(invited) = 21, n(responses) = 19; Varde in Oct–Nov 2013, n(invited) = 11, n(responses) = 11). The second highest response rate was found in the post-intervention measurement (Kolding in Apr–May 2014, n(invited) = 19, n(response) = 12; Varde in Jul–Sep 2014, n(invited) = 11, n(response) = 8). The lowest response rate was found in the 12-month follow-up (Kolding in May 2015, n(invited) = 19, n(response) = 11; Varde in May 2015, n(invited) = 11, n(response) = 7). The main reason for dropout was the long time period between the pre-intervention measurement and the 12-month follow-up measurement. Several participants had changed positions or organisations within this time period. Other reasons for dropout were holidays or, in the case of the 12-month measurement, difficulty relating to the topic [13].

In general, the relatively low number of participants made it difficult to carry out statistical analysis, and thus the results and their interpretation had to be produced based on descriptive cross-sectional results and with the maximum number of participants at each point of measurement.

The data were analyzed separately for the two municipalities and divided into the three points of measurement. For each question answered on the five-point Likert scale, a mean was calculated. Answers using the ‘do not know’ option were counted as missing values.

The text written in the open text boxes was included in the qualitative text analysis described below.

### Data – qualitative interviews

Qualitative interviews were performed with one representative from each administrative department in both municipalities (Kolding *n* = 8, Varde = 6). The informants were all part of the interventions throughout the whole period. The interviews were part of the needs assessments and were therefore conducted before the intervention period started. Thus, the qualitative data cannot be used to explain the possible changes of the interventions, but instead help to describe and understand the perspectives and perceptions of the informants related to EIPM in HEPA policy development.

A semi-structured interview guide directed the interviews. The main themes from the interviews reported in this paper were the use of research/stakeholder/target group knowledge, determinants for use of research/stakeholder/target group knowledge, and political request for use of research knowledge. In addition to these themes, the interview guide included questions related to intersectoral collaboration.

The interviews were recorded, transcribed and the text was analyzed by deductive qualitative content analysis [28]. Therefore, texts supporting or opposing the findings from the pre-intervention questionnaire was highlighted. In this paper, selected statements will be presented in order to assist with reflection on the quantitative measures.

## Results

### Quantitative data

The questionnaire results are summarised in Table 3. They are presented separately for the two municipalities and divided into the three points of measurement. For each measurement, the number of respondents (n) and a mean score for each item on the five-point Likert scale are given. All of the following descriptions refer to changes in means. References are made to Table 3 by letters.

**Table 3:** Research knowledge, stakeholder knowledge and target group knowledge in the two municipalities at pre-intervention, post-intervention and 12-month follow-up; numbers of respondents and means of the responses (scale 1–5 (1: to a very low degree; 2: to a low degree; 3: neither to a low nor high degree; 4: to a high degree; 5: to a very high degree) and a ‘do not know’ option (counted as missing)

	Kolding						Varde					
Item	Pre		Post		12 m follow-up		Pre		Post		12 m follow-up	
Research knowledge	n	Mean	n	Mean	n	Mean	n	Mean	n	Mean	n	Mean
Search for knowledge from research	19	3.6 (A)	10	3.2 (A)	11	3.3 (A)	8	3.6 (B)	7	4.0 (B)	7	4.1 (B)
Translation to local needs	19	3.5 (C)	9	3.6 (C)	11	3.6 (C)	10	3.5 (D)	8	3.8 (D)	6	4.2 (D)
Political request for use	18	1.9 (E)	10	2,6 (E)	11	2.5 (E)	6	3.7 (F)	7	3.7 (F)	6	4.7 (F)
Organisational procedures	19	2.2 (G)	10	2.6 (G)	10	2.6 (G)	9	2.6 (H)	7	2.7 (H)	6	2.8 (H)
Influence on final decisions	17	2.9 (I)	10	2.8 (I)	11	2.5 (I)	8	3.3 (J)	8	3.5 (J)	5	3.8 (J)
Stakeholder knowledge												
Collection of knowledge from internal stakeholders	19	3.5 (K)	9	3.6 (K)	11	4.0 (K)	10	4.0 (L)	8	3.5 (L)	5	4.4 (L)
Collection of knowledge from external stakeholders	19	3.5 (M)	9	3.8 (M)	11	3.9 (M)	10	3.8 (N)	8	3.3 (N)	5	4.0 (N)
Internal stakeholder knowledge influence on final decisions	19	3.0 (O)	9	3.6 (O)	10	3.5 (O)	10	3.8 (P)	8	4.0 (P)	5	4.0 (P)
External stakeholder knowledge influence on final decisions	19	3.3 (Q)	9	3.6 (Q)	10	3.3 (Q)	10	2.9 (R)	8	3.5 (R)	5	4.0 (R)
Organisational procedures	17	2.9 (S)	9	3.2 (S)	11	3.6 (S)	8	3.5 (T)	7	2.7 (T)	5	3.0 (T)
Target group Knowledge												
Access to knowledge of characteristics of target group	18	3.3 (U)	9	3.2 (U)	11	3.6 (U)	9	3.8 (V)	7	4.0 (V)	4	4.0 (V)
Target group characteristics influence on final decisions	18	3.3 (W)	9	3.7 (W)	11	3.9 (W)	9	3.8 (X)	8	3.9 (X)	4	4.0 (X)
Access to knowledge of needs and values of target group	19	2.9 (Y)	8	3.4 (Y)	11	3.5 (Y)	9	3.8 (Z)	8	3.6 (Z)	6	3.7 (Z)
Organisational procedures	17	2.2 (A1)	9	2.9 (A1)	11	3.4 (A1)	9	3.2 (B1)	7	2.7 (B1)	6	3.2 (B1)
Influence on final decisions	19	3.4 (C1)	9	3.4 (C1)	11	3.8 (C1)	9	3.7 (D1)	6	4.0 (D1)	5	4.2 (D1)

Letters in brackets are used to refer to the supplementary text in the results section

#### Conceptual knowledge use

In Kolding, the collection of knowledge from both internal and external stakeholders, and access to knowledge on needs and values of target groups increased during the whole follow-up period (K, M, Y). A decrease in the search for knowledge from research (A) and access to knowledge of target group characteristics (U) was seen after the intervention; however, an increase occurred again 12 months later, surpassing the pre-intervention level for knowledge of target-group characteristics. In Varde, the situation was different because an increase in the search for knowledge from research (B), and an increase in access to knowledge of target group characteristics (V), was seen during the whole follow-up period. At the same time, collection of knowledge from both internal and external stakeholders decreased during the intervention period, but an increase occurred again 12 months later to above the pre-intervention level (L, N).

#### Knowledge translation

In Kolding, the assessment of how easily knowledge from research can be translated into local needs increased slightly during the intervention period and remained at that level 12 months later (C). In Varde, the same was seen after the intervention, and a further increase was found 12 months later (D).

#### Request for research knowledge

In Kolding, the experience of political request for knowledge use increased during the intervention period but decreased slightly 12 months later (E). In Varde, it was different because the request for knowledge remained unchanged after the intervention but increased quite a lot 12 months later (F).

#### Organisational procedures

In Kolding, the level at which organisational procedures ensured the use of relevant knowledge from research in policy development increased after the intervention and remained at that level 12 months later (G). Additionally, the use of stakeholder and target group knowledge increased during the whole follow-up period (S, A1). In Varde, the level of organisational procedures in relation to knowledge from research increased during the whole follow-up period (H). For stakeholder and target group knowledge, it decreased after the intervention, but an increase was seen 12 months later; for target group knowledge, the increase was to the pre-intervention level (T, B1).

#### Instrumental knowledge use

In Kolding, the influence of research knowledge on final policy decisions decreased during the whole follow-up period (I). In contrast, for knowledge from internal stakeholders and knowledge of target groups, it increased in the same period (O, W). The influence of knowledge from external stakeholders increased during the intervention period, but after 12 months, it decreased to the pre-intervention level (Q). In Varde, another tendency was found because the influence of knowledge from research, from external stakeholders, and on target groups increased during the whole follow-up period (J, N, X). For internal stakeholders, it also increased during the intervention period but fell back to the pre-intervention level after 12 months (P).

### Qualitative data

The statements from the qualitative interviews and the open text boxes in the questionnaires are presented in Table 4. The statements are marked to show which part of the data they belong to. For example, (V, pre) indicates that the statement comes from the pre-measurement questionnaire in Varde, and (K, int) indicates that it comes from interviews in Kolding. The statements are also numbered and referred to in the following analysis.

**Table 4:** Statements from interviews (int) and open text boxes in questionnaires (pre, post, 12 m) on use of research/stakeholder/target group knowledge, determinants for use of research/stakeholder/target group knowledge and political request for use of knowledge from Kolding (K) and Varde (V)

Themes	Statements
Use of research knowledge	(1) On top of my head I would say that knowledge from research is prioritised. It is at least something I try to base my work on, when I start new projects (K, pre)
	(2) It is not natural for the system to work with research-based knowledge (V, int)
	(3) It is not like 20 years ago, where we just started – it was easier then. Sometimes we get too academic. It’s really a balance (K, int)
	(4) In order for you to reach something in a society that is as built up as the Danish, you need to have that evidence-based research (K, int)
	(5) We are constantly searching. However, it is not all we need that exists. I think that in a public official’s argumentation for how we should do things in a political context, we need to have the professionalism that is supported by the research that can be found (V, int)
Determinates for use of research knowledge	(6) To find out what’s in the field, you need to be really proactive. You can of course search on Google and read the pole up and down. Time is a big barrier (K, int)
	(7) There is a culture within the health area – because many employees and managers have a background in public health science, so it is normal to search for available knowledge from research in regard to various activities (K, pre)
	(8) There is a great difference in the cultures of the individual departments (V, int)
	(9) It is of course dependent on timeline and deadline (K, pre)
	(10) It depends a great deal on the subject and how you relate to the content in the research based knowledge (K, pre)
	(11) It is probably very much dependent on individuals whether knowledge from research is integrated more than procedures (V, pre)
	(12) The intervention did absolutely help to increase the academic standard of the product outcome. More time was spent in examining research within the field. Experiences are transferred to other fields where there is work with policies (V, 12 m)
	(13) The intervention has supported the importance of the research-based approach – methods, which we have applied in developing other strategies (V, 12 m)
	(14) I have some good people around me. But, we may not be good enough for that, I will say, even though I subscribe to many journals (K, int)
	(15) I think our accreditation process is a way to get the research-based knowledge under the skin (K, int)
	(16) First of all, I’m hiring newly educated academics who are used to searching literature when they need to start something new (V, int)
Use of stakeholder knowledge	(17) There are, for example, procedures for hearings (V, pre)
	(18) There is often talk about establishing working groups with external as well as internal stakeholders in connection to developing a new policy or strategy (K, pre)
	(19) We try to involve them by holding some meetings, theme meetings or the like, and it is a challenge to get people to participate
	(20) If it is going to be fast, we will typically be based on practice. We will ask the employees how they experience it (K, int)
Determinates for use of stakeholder knowledge	(21) If we do not use stakeholder knowledge and interest, then we have no authority (K, int)
Use of target group knowledge	(22) If we are in a hurry, we will look at the statistics we already have and maybe make a call if we know that we can quickly find what we need there. But, then we do not have time to go out and make it big. Then, it is the statistics and our experience, we lean on (K, int)
	(23) No, once we have made a new health policy, we have invited some different stakeholders. We have not made a whole lot of citizen involvement, and we do not think that it would contribute a lot (K, int)
Political request for use of knowledge	(24) Often we start working partly with knowledge based on research, but then it gets ‘override/overruled’ by political preferences, which are then prioritised (K, pre)
	(25) Our experience is that it highly depends on the politicians who set the agenda, and here attitudes are prominent, not research-based knowledge and evidence, which are most important (K, post)
	(26) No, there is always political pressure and an appeal for user involvement, which ‘overrides’ the will to start a process based on research knowledge (K, 12 m)
	(27) Often, our experience is that external stakeholder involvement is organisational and politically requested, but not always wanted to be used in the end (K, 12 m)
	(28) Where we have used evidence, we will always write it into our case making, and I find that politicians listen more and more to the research-based evidence
	(29) It is not the case that they themselves are saying that someone has researched something and ask if we can investigate it a bit closer (K, int)
	(30) We are in a political system and sometimes I think if good knowledge will give better political decisions? You can sometimes question that (K, int)
	(31) Politicians would like to feel and believe. And sometimes they get a little scared when we come and say that is a fact. It does not always fit into their political agenda to get the facts on the table. So it is double. On the other hand, they would like to appear as someone basing their policy on facts and therefore they are happy if we can deliver it (K, int)

The qualitative data indicated that using knowledge – from all three sources – is generally perceived as a working method to strive for in the municipalities, especially when starting up new projects (statements 1, 5, 7). However, one informant noted that it was easier to develop policies and interventions 20 years ago and that the policy process today can become too academic (statement 3).

Using knowledge was not always doable in the municipal working culture, mainly due to time limits and cultural factors (statements 6–16). The informants mentioned that it is not natural for them to work with knowledge from research, and it can be hard to find what they need (statement 5). There were perceived differences among departments in the municipalities, e.g. the health departments were understood to be more trained and experienced in searching for knowledge compared with other departments (statement 7). It is also indicated that newly educated academics are often the best at this (statement 16). The informants believed that the level of knowledge use in a policy process is very dependent on the individuals involved (statements 11, 14).

The informants mentioned processes such as public hearings as a way of including stakeholder knowledge in policy development (statement 17). They also revealed that they try to invite stakeholders to participate in working groups and meetings but that it is often difficult to bring them on board (statement 18, 19). Regarding target group knowledge, its use seemed to be mostly based on the statistics that the municipality already had in place, especially if the staff were in a hurry (statement 22). One informant questioned whether including citizens in policy development would contribute anything (statement 23). On the other hand, another informant stated that, if the municipality does not use knowledge and interests from stakeholders, then it has no authority (statement 21).

In general, the informants believed that the politicians in the municipality preferred basing their work on values and interests, primarily taking into account knowledge from stakeholders and target groups (statements 25, 26). Often, the informants had started a policy process that included knowledge from research, but then this knowledge was overruled by political preferences (statement 24). On the other hand, the informants thought that politicians liked to base their policies on knowledge from research; therefore, they appreciate when the departments can deliver this – especially if it fits to their already specified agendas (statement 31). Furthermore, one informant felt that the politicians were more open to suggestions from the departments if those suggestions were based on knowledge from research (statement 28).

In the 12-month follow-up, informants recognised that the contextually tailored intervention increased the academic standard of the HEPA policy that was developed because more time than normal was spent on finding and using knowledge from research during the policy development process. They noted that this experience was transferred to other fields working on policy development. Furthermore, they mentioned future accreditation processes as possible facilitators for EIPM (statements 12, 13).

## Discussion

Several important changes occurred in both municipalities. One was the improvement in organisational procedures for the use of research knowledge seen in the increased Likert scale mean score and reported in the 12-month follow-up questionnaire (see Table 4, statement 13). This is an important change since the issue that the EIPM level is often dependent only on individual characteristics of the persons involved needs to be overcome [14, 26, 29, 30]. Another important change was the increased political request for knowledge use in general, as this is a central facilitator for EIPM [16].

In both municipalities, it was considered easier to transfer research knowledge to local needs after the intervention. This was the third most important change because – in HEPA and other public health and health-promotion related topics – the ability to use knowledge from research in local settings is highly important [16, 31].

There were some differences in the results of the two municipalities in relation to the changes in their use of the three different types of knowledge. Based on this study, there is no solid evidence for explaining these differences, hence the explanations below are only suggestions.

The fact that, in Kolding, stakeholder and target group knowledge increased but research knowledge decreased could be attributed to the intervention in this municipality being focused very much on intersectoral collaboration and hence on knowledge from different departments and collaborators. It is interesting to note that the interviews performed prior to the intervention showed that one informant did not really see the value of including target groups in the policy development (see Table 4, statement 23). It would have been valuable to follow-up on that statement after the intervention.

In Varde, where the search for research knowledge increased during the whole follow-up period, the intervention focused more on developing a structure for EIPM. Hence, there was a greater focus on research knowledge. Another reason might be the fact that the participants in Kolding were in more senior positions and were familiar with the use of research evidence, whereas the participants in Varde were in more junior positions and thus had greater potential for increasing their perception of research evidence use. On the other hand, junior staff members are often, due to their educational background, quite familiar with the relevance of research knowledge and how they can find it. In Varde, the Health and Rehabilitation Department (those responsible for policy development) already had quite a strong focus on the use of research knowledge due to prior collaborations with research institutions. They might have already had a more research-minded mind-set from the beginning, which gave them a head start.

The qualitative results suggested that EIPM is very dependent on individuals and individual factors. For future research it would be very interesting to perform further studies on person-level determinants and possibly define categories of individuals that can enable EIPM.

In several components, even if an increase was seen after the intervention, there was a minor decrease during the 12-month follow-up, though mostly not to the pre-intervention level. Overall, it seems to be a challenge to sustain the developments achieved during the interventions. Continuous partnering with researchers and booster sessions after the interventions might be methods for tackling this.

The interventions were a novel way to study policy-making by ‘jumping on the wagon’ so that real policy cases and real policy-makers from different departments in local contexts could participate in the interventions together with researchers.

The results are descriptive, and therefore generalisation is not feasible. However, it can be stated that the contextually tailored interventions seemed to enhance EIPM in many of its dimensions; further, even though some of the findings were not sustainable in the longer run, there were indications that the interventions were only the beginning of more sustainable organisational structures for EIPM.

Based on these preliminary results, it is suggested that more and larger-scale research on the contextually tailored interventions for EIPM – not only within the area of HEPA but also in other lifestyle-related topics – be developed and implemented.

### Limitations of the study

This study had few participants and a relatively high drop-out rate for the post-intervention and the 12-month follow-up measurements, and therefore only descriptive statistical methods could be used. Furthermore, the interventions took place in their natural contexts, meaning that other factors, such as related projects and activities in the municipalities, might have affected the observed changes. Thus, the results need to be interpreted carefully and should only be seen as indicative findings in need of further confirmation with larger samples.

## Conclusion

Despite methodological limitations, the results of this intervention study in two Danish municipalities showed that contextually tailored interventions have the potential to increase EIPM in HEPA policies in relation to finding and using different types of knowledge, to translate knowledge to local contexts, to develop procedures for knowledge use and to increase political request for use of knowledge in policy development. Sustainability of the changes is a challenge, which must be taken into account in future studies.

## Additional file

Additional file 1: Questionnaire used in the study reported in the paper. Questions on a light grey background are not included in the analysis in this paper. (DOCX 26 kb)

## Abbreviations

EIPM: evidence-informed policy-making
HEPA: health-enhancing physical activity
