# Supplementary material for: Contextually tailored interventions can increase evidence-informed policy-making on health-enhancing physical activity: the experiences of two Danish municipalities
Source: Health Res Policy Syst. 2018 Feb 21;16:14. doi: 10.1186/s12961-018-0290-4 (PMC5822477; doi:10.1186/s12961-018-0290-4)
Supplement: Supplementary file 1 — Questionnaire used in the study reported in the paper. Questions on a light grey background are not included in the analysis in this paper. (DOCX 26 kb) [file 12961_2018_290_MOESM1_ESM.docx]

**Additional file**

**Questionnaire used in the study reported in the paper “Contextually tailored interventions can increase evidence-informed policymaking on health-enhancing physical activity: The experiences of two Danish municipalities”. Questions on a light grey background are not included in the analysis in this paper.**

| **Questions** | **Follow-up questions** |
| --- | --- |
| **THE FOLLOWING QUESTIONS DEAL WITH HOW YOUR ORGANIZATION WORKS WITH THE USE OF KNOWLEDGE FROM RESEARCH** | |
| To what extent is relevant knowledge from research being searched for when working with **policy development** in your organization? (E.g. in internet based databases, via contacting a research organization etc.)  1: To a very low degree  2: To a low degree  3: Neither to a low or high degree  4: To a high degree  5: To a very high degree  **Policy development:** By policy development we mean the process from the first initiatives to a concrete proposal is formulated.  **Knowledge from research:**  By knowledge from research we mean knowledge provided through scientific methods | If relevant, please give examples of how your organization is searching for relevant knowledge from research: |
| To what extent can knowledge from research be easily **translated** to local needs?  1: To a very low degree  2: To a low degree  3: Neither to a low or high degree  4: To a high degree  5: To a very high degree  **Translate:** By translate we mean how to make knowledge from research relevant in relation to the development of a specific policy in the local context. | If relevant, please elaborate on your answer: |
| To what extent do you experience **barriers** for the use of knowledge from research when working with **policy development** in your organization?  1: To a very low degree  2: To a low degree  3: Neither to a low or high degree  4: To a high degree  5: To a very high degree  **Barriers:** By barriers we mean factors limiting the use of evidence.  **Policy development:** By policy development we mean the process from the first initiatives to a concrete proposal is formulated. | If relevant, which types of barriers do you experience? (Multiple answers possible)   - Lack of time - Lack of awareness on knowledge from research - Lack of competences and experiences - Lack of or difficult access to relevant knowledge from research - Lack of economic resources - Lack of organizational procedures ensuring the use of knowledge from research - Lack of tradition for the use of knowledge from research - Lack of political request for the use of knowledge from research - Lack of collaboration between researchers and decision makers - Knowledge from research is difficult to translate to local needs - Other barriers (please mention which types) _____________________ |
| To what extent do you experience factors promoting the use of knowledge from research when working with **policy development** in your organization?  1: To a very low degree  2: To a low degree  3: Neither to a low or high degree  4: To a high degree  5: To a very high degree  **Policy development:** By policy development we mean the process from the first initiatives to a concrete proposal is formulated. | If relevant, which types of promoting factors do you experience? (multiple answers possible)   - Sufficient time - Sufficient awareness on knowledge from research - Sufficient competences and experiences - Sufficient access to knowledge from research - Sufficient economic resources - Organizational procedures for the use of knowledge from research - Organizational culture supporting the use of knowledge from research - Political request for the use of knowledge from research - Sufficient collaboration between researchers and decision makers - Sufficient access to knowledge which can be translated to local needs - Other facilitators (please mention which types) ________________ |
| To what extent do politicians in your organization request use of knowledge from research in **policy development**?  1: To a very low degree  2: To a low degree  3: Neither to a low or high degree  4: To a high degree  5: To a very high degree  **Policy development:** By policy development we mean the process from the first initiatives to a concrete proposal is formulated. |  |
| To what extent does your organization have **procedures** ensuring the use of relevant knowledge from research in **policy development**?  1: To a very low degree  2: To a low degree  3: Neither to a low or high degree  4: To a high degree  5: To a very high degree  **Procedures:** By procedures we mean established routines or guidelines for the identification and use of knowledge from research.  **Policy development:** By policy development we mean the process from the first initiatives to a concrete proposal is formulated. | If relevant, please describe the procedures: |
| To what extent does knowledge from research **influence** the **final decisions** made in your organizations?  1: To a very low degree  2: To a low degree  3: Neither to a low or high degree  4: To a high degree  5: To a very high degree  **Influence:** By influence we mean when knowledge from research affects the contents of the policies – i.e. when it is likely that the content of the policy would have been different without including knowledge from research in the process.  **Final decisions:** By final decisions we mean decisions and policies adopted by political committees in your organization |  |
| **THE FOLLOWING QUESTIONS DEAL WITH HOW YOUR ORGANIZATION WORKS WITH THE USE OF KNOWLEDGE FROM VARIOUS STAKEHOLDERS** | |
| To what extent does your organization collect knowledge from relevant *internal* stakeholders (from other departments/sectors, practitioners at other levels of the organization) when working with **policy development?**  1: To a very low degree  2: To a low degree  3: Neither to a low or high degree  4: To a high degree  5: To a very high degree  6: Do not know  **Policy development:** By policy development we mean the process from the first initiatives to a concrete proposal is formulated.  **Stakeholders:** By stakeholders we mean organizations, groups of persons or individuals who are influencing or are influenced by the dispositions made in your organization. Knowledge from stakeholders covers contextual knowledge developed through the practical experiences of the stakeholders. | If relevant, please give examples of the procedures for involvement of internal stakeholders: |
| To what extent does your organization collect knowledge from relevant *external* stakeholders (e.g. voluntary organizations, commercial organizations) when working with **policy development**?  1: To a very low degree  2: To a low degree  3: Neither to a low or high degree  4: To a high degree  5: To a very high degree  6: Do not know  **Policy development:** By policy development we mean the process from the first initiatives to a concrete proposal is formulated. | If relevant, please give examples of the procedures for involvement of external stakeholders: |
| There can be barriers connected to the use of knowledge from various stakeholders when working with **policy development**. To what extent do you experience such barriers in your organization?  1: To a very low degree  2: To a low degree  3: Neither to a low or high degree  4: To a high degree  5: To a very high degree  6: Do not know  **Barriers:** By barriers we mean factors limiting or restricting the use of knowledge from stakeholders  **Policy development:** By policy development we mean the process from the first initiatives to a concrete proposal is formulated. | If relevant, which types of barriers do you experience? (multiple answers possible)   - Lack of time - Lack of awareness of knowledge from stakeholders - Lack of competences and experiences - Lack of or difficult access to relevant knowledge from stakeholders - Lack of economic resources - Lack of organizational procedures for including knowledge from stakeholders - Lack of tradition for inclusion of knowledge from stakeholders - Lack of political request for inclusion of knowledge from stakeholders - Lack of collaboration between stakeholders and decision makers - Other barriers (please mention which types) _____________________ |
| To what extent do you experience factors promoting the inclusion of knowledge from various stakeholders when working with **policy development** in your organization?  1: To a very low degree  2: To a low degree  3: Neither to a low or high degree  4: To a high degree  5: To a very high degree  6: Do not know  **Policy development:** By policy development we mean the process from the first initiatives to a concrete proposal is formulated. | If relevant, which types of promoting factors do you experience? (multiple answers possible)   - Sufficient time - Sufficient awareness of knowledge from stakeholders - Sufficient competences and experiences - Sufficient access to knowledge from stakeholders - Sufficient economic resources - Organizational procedures for the use inclusion of knowledge from stakeholders - Organizational culture supporting inclusion of knowledge from stakeholders - Political request for the inclusion of knowledge from stakeholders - Sufficient collaboration between stakeholders and decision makers - Other factors (please mention which types) ________________ |
| To what extent does knowledge from *internal* stakeholders (e.g. from other departments/sectors, practitioners at other levels of the organization) **influence** the **final decisions** made in your organizations?  1: To a very low degree  2: To a low degree  3: Neither to a low or high degree  4: To a high degree  5: To a very high degree  6: Do not know  **Influence:** By influence we mean when knowledge from internal stakeholders affects the contents of the policies and when it is likely that the content of the policy would have been different without including knowledge from internal stakeholders in the process.  **Final decisions:** By final decisions we mean decisions and policies adopted by by political committees in your organization |  |
| To what extent does knowledge from *external* stakeholders (e.g. voluntary organizations, commercial organizations) **influence** the **final decisions** made in your organizations?  1: To a very low degree  2: To a low degree  3: Neither to a low or high degree  4: To a high degree  5: To a very high degree  6: Do not know  **Influence:** By influence we mean when knowledge from external stakeholders affects the contents of the policies – i.e. when it is likely that the content of the policy would have been different without including knowledge from internal stakeholders in the process.  **Final decisions:** By final decisions we mean decisions and policies adopted by political committees in your organization |  |
| To what extent does your organization have **procedures** ensuring the inclusion of knowledge from various stakeholders in **policy development**?  1: To a very low degree  2: To a low degree  3: Neither to a low or high degree  4: To a high degree  5: To a very high degree  6: Do not know  **Procedures:** By procedures we mean guidelines or established routines for the identification and use of knowledge from various stakeholders.  **Policy development:** By policy development we mean the process from the first initiatives to a concrete proposal is formulated. |  |
| To what extent is there a search for relevant knowledge from prior similar policy initiatives (e.g. from evaluations, best practice etc.) when working with **policy development** in your organization?  1: To a very low degree  2: To a low degree  3: Neither to a low or high degree  4: To a high degree  5: To a very high degree  6: Do not know |  |
| To what extent does knowledge from prior similar policy initiatives (E.g. from evaluations, best practice etc.) **influence** the final decisions made in your organizations?  1: To a very low degree  2: To a low degree  3: Neither to a low or high degree  4: To a high degree  5: To a very high degree  6: Do not know  **Influence:** By influence we mean when knowledge from external stakeholders affects the contents of the policies and when it is likely that the content of the policy would have been different without including knowledge from external stakeholders in the process |  |
| To what extent does knowledge on political agendas and interests influence the work with **policy development** in your organization? ‘’  1: To a very low degree  2: To a low degree  3: Neither to a low or high degree  4: To a high degree  5: To a very high degree  6: Do not know  **Policy development:** By policy development we mean the process from the first initiatives to a concrete proposal is formulated. | If relevant, please elaborate on your answer: |
| **THE FOLLOWING QUESTIONS DEAL WITH HOW YOUR ORGANIZATION WORKS WITH KNOWLEDGE ON TARGET GROUPS** | |
| To what extent does your organization have **access** to knowledge on the **characteristics of target groups** in focus in each policy development (E.g. through statistics, own data collections etc.)?  1: To a very low degree  2: To a low degree  3: Neither to a low or high degree  4: To a high degree  5: To a very high degree  6: Do not know  **Access:** By access we mean when knowledge on characteristics of target groups is available for you in the work process  **Characteristics of target groups:** By characteristics of target groups we mean objective factors characterizing a given target group (e.g. the proportion of overweight persons, housing conditions etc.) |  |
| To what extent does knowledge on target group characteristics **influence** the **final decisions** made in your organization?  1: To a very low degree  2: To a low degree  3: Neither to a low or high degree  4: To a high degree  5: To a very high degree  6: Do not know  **Influence:** By influence we mean when knowledge on target groups affects the contents of the policies and when it is likely that the content of the policy would have been different without including knowledge on target groups in the process.  **Final decisions:** By final decisions we mean decisions and policies adopted by political committees in your organization |  |
| To what extent does your organization have **access** to knowledge on needs and values of target groups for the policy you are working with?  1: To a very low degree  2: To a low degree  3: Neither to a low or high degree  4: To a high degree  5: To a very high degree  6: Do not know  **Access:** By access we mean when knowledge on needs, and values is available for you in the work process. |  |
| To what extent does your organization have **procedures** ensuring the use of knowledge on needs and values of target groups in the policy development process?  1: To a very low degree  2: To a low degree  3: Neither to a low or high degree  4: To a high degree  5: To a very high degree  6: Do not know  **Procedures**: By procedures we mean guidelines or established routines for the identification and use of knowledge on needs and values of target groups |  |
| There can be **barriers** connected to the inclusion of knowledge on needs and values of target groups when working with **policy development**? To what extent do you experience this in your organization?  1: To a very low degree  2: To a low degree  3: Neither to a low or high degree  4: To a high degree  5: To a very high degree  6: Do not know  **Barriers:** By barriers we mean factors limiting or restricting the use of evidence  **Policy development:** By policy development we mean the process from the first initiatives to a concrete proposal is formulated. | If relevant, which types of barriers do you experience? (multiple answers possible)   - Lack of time - Lack of awareness of knowledge on needs and values of target groups - Lack of competences and experiences - Lack of or difficult access to knowledge on needs and values of target groups - Lack of economic resources - Lack of organizational procedures for including knowledge on needs and values of target groups - Lack of tradition for inclusion of knowledge on needs and values of target groups - Lack of political request for inclusion of knowledge on needs and values of target groups - Lack of involvement from the target groups themselves - Other barriers (please mention which types) _____________________ |
| To what extent do you experience factors promoting the use of knowledge on needs and values of target groups when working with **policy development**?  1: To a very low degree  2: To a low degree  3: Neither to a low or high degree  4: To a high degree  5: To a very high degree  6: Do not know  **Policy development:** By policy development we mean the process from the first initiatives to a concrete proposal is formulated. | If relevant, which types of promoting factors do you experience? (multiple answers possible)   - Sufficient time - Sufficient awareness on use of knowledge on needs and values of target groups - Sufficient competences and experiences - Sufficient access to knowledge on needs and values of target groups - Sufficient economic resources - Organizational procedures for the use inclusion of knowledge on needs and values of target groups - Political request for the inclusion of knowledge on needs and values of target groups - Sufficient collaboration between target groups and decision makers - Other factors (please mention which types) ________________ |
| To what extent does knowledge on needs, values and preferences of the target group **influence** the **final decisions** made in your organization?  1: To a very low degree  2: To a low degree  3: Neither to a low or high degree  4: To a high degree  5: To a very high degree  6: Do not know  **Influence:** By influence we mean when knowledge from external stakeholders affects the contents of the policies, and when it is likely that the content of the policy would have been different without including knowledge from external stakeholders in the process  **Final decisions:** By final decisions we mean decisions and policies adopted by political committees in your organization |  |
| **THE FOLLOWING QUESTIONS DEAL WITH THE USE OF DIFFERENT TYPES OF KNOWLEDGE IN YOUR ORGANIZATION** | |
| Which of the following sources of knowledge have the greatest influence on the **final decisions** made in your organizations:  (label the sources with numbers: 1: the greatest influence, 5: the least influence)     - **Knowledge from research** - Knowledge from **stakeholders** - Knowledge on **target group characteristics** - Knowledge on needs and values of target groups - Knowledge on political agendas and interests   **Final decisions:** By final decisions we mean decisions and policies adopted by/passed by political committees in your organization  **Knowledge from research:**  By knowledge from research we mean knowledge provided through scientific methods  **Stakeholders:** By stakeholders we mean organizations, groups of persons or individuals who are influencing or are influenced by the dispositions made in your organization. Knowledge from stakeholders covers contextual knowledge developed through the practical experiences of the stakeholders.  **Target group characteristics:** By target group characteristics we mean objective factors characterizing a given target group (e.g. the proportion of overweight persons, housing conditions etc.) |  |
| Which of the following sources of knowledge *should ideally* have the greatest influence on the **final decisions** made in your organizations:  (label the sources with numbers: 1: the greatest influence, 5: the least influence)     - **Knowledge from research** - Knowledge from **stakeholders** - Knowledge on **target group characteristics** - Knowledge on needs and values of target groups - Knowledge on political agendas and interests   **Final decisions:** By final decisions we mean decisions and policies adopted by/passed by political committees in your organization  **Knowledge from research:**  By knowledge from research we mean knowledge provided through scientific methods  **Stakeholders:** By stakeholders we mean organizations, groups of persons or individuals who are influencing or are influenced by the dispositions made in your organization. Knowledge from stakeholders covers contextual knowledge developed through the practical experiences of the stakeholders.  **Target group characteristics:** By target group characteristics we mean objective factors characterizing a given target group (e.g. the proportion of overweight persons, housing conditions etc.) |  |
| To what extent are you satisfied with the level of **knowledge use** in the policy processes in your organization?  1: To a very low degree  2: To a low degree  3: Neither to a low or high degree  4: To a high degree  5: To a very high degree  6: Do not know  **Knowledge use**: By knowledge use we mean the aggregated use of knowledge from research, knowledge from stakeholders and knowledge on target groups. | If relevant, please elaborate on your answers: |
| If you have any further comments in relation to the use of different types of knowledge when working with policy development in your organization, please write them here: _______________________________ |  |
| **FINALLY, WE WOULD LIKE TO ASK YOU A FEW QUESTIONS ON PERSONAL DETAILS** | |
| Gender   - Female - Male |  |
| How old are you?  (Age in years) _____________________________ |  |
| In which department/section are you employed?  ________________________________ | If this information is available beforehand you can put it in the background scheme instead. |
| For have long have you been employed in this department/section? (Number of years) |  |
| Which position do you hold? | If this information is available beforehand you can put it in the background scheme instead. |
| For how long have you held your current position in your organization? (Number of years) |  |
| What is the highest level of education that you have completed? (please tick the  highest level you have completed)   - Primary and secondary school - Basic vocational courses, technical college etc. - High School - Vocational education - Short further education 1-2 years) - Medium long further education 3-4 years - Long further education 5 years and more - Do not wish to answer | Here the Danish scale is used as an example, but you should use the education scale most commonly known in your country. On the basis of these different scales we will do a coding in the final dataset – creating a common variable for educational level. |
